# Physical activity assessment by accelerometry in people with heart failure

**DOI:** 10.1186/s13102-020-00196-7

**Published:** 2020-08-12

**Authors:** Grace O. Dibben, Manish M. Gandhi, Rod S. Taylor, Hasnain M. Dalal, Brad Metcalf, Patrick Doherty, Lars H. Tang, Mark Kelson, Melvyn Hillsdon

**Affiliations:** 1 University of Exeter Medical School, Knowledge Spa, Royal Cornwall Hospitals NHS Trust, Truro, UK; 2 Department of Cardiology, Royal Devon & Exeter Hospital, Exeter, UK; 3 Institute of Health Research (Primary Care), University of Exeter Medical School, St. Luke’s Campus, Exeter, UK; 4 Institute of Health and Wellbeing, University of Glasgow, Glasgow, UK; 5 Department of Sport and Health Sciences, University of Exeter, St Luke’s Campus, Exeter, UK; 6 Department of Health Sciences, University of York, York, UK; 7 National Centre for Rehabilitation and Palliative Care, University of Southern Denmark and Odense University Hospital, Ringsted, Denmark; 8 Department of Physiotherapy and Occupational Therapy, Næstved-Slagelse-Ringsted Hospitals, Region Zealand, Denmark; 9 Department of Regional Health Research, University of Southern Denmark, Odense, Denmark; 10 Department of Mathematics, College of Engineering, Mathematics and Physical Sciences, University of Exeter, Exeter, UK

**Keywords:** Heart failure, Accelerometer, Physical activity, Cut-points, Activity intensity

## Abstract

**Background:**

International guidelines for physical activity recommend at least 150 min per week of moderate-to-vigorous physical activity (MVPA) for adults, including those with cardiac disease. There is yet to be consensus on the most appropriate way to categorise raw accelerometer data into behaviourally relevant metrics such as intensity, especially in chronic disease populations. Therefore the aim of this study was to estimate acceleration values corresponding to inactivity and MVPA during daily living activities of patients with heart failure (HF), via calibration with oxygen consumption (VO_2_) and to compare these values to previously published, commonly applied PA intensity thresholds which are based on healthy adults.

**Methods:**

Twenty-two adults with HF (mean age 71 ± 14 years) undertook a range of daily living activities (including laying down, sitting, standing and walking) whilst measuring PA via wrist- and hip-worn accelerometers and VO_2_ via indirect calorimetry. Raw accelerometer output was used to compute PA in units of milligravity (mg). Energy expenditure across each of the activities was converted into measured METs (VO_2_/resting metabolic rate) and standard METs (VO_2_/3.5 ml/kg/min). PA energy costs were also compared with predicted METs in the compendium of physical activities. Location specific activity intensity thresholds were established via multilevel mixed effects linear regression and receiver operator characteristic curve analysis. A leave-one-out method was used to cross-validate the thresholds.

**Results:**

Accelerometer values corresponding with intensity thresholds for inactivity (< 1.5METs) and MVPA (≥3.0METs) were > 50% lower than previously published intensity thresholds for both wrists and waist accelerometers (inactivity: 16.7 to 18.6 mg versus 45.8 mg; MVPA: 43.1 to 49.0 mg versus 93.2 to 100 mg). Measured METs were higher than both standard METs (34–35%) and predicted METs (45–105%) across all standing and walking activities.

**Conclusion:**

HF specific accelerometer intensity thresholds for inactivity and MVPA are lower than previously published thresholds based on healthy adults, due to lower resting metabolic rate and greater energy expenditure during daily living activities for HF patients.

**Trial registration:**

Clinical trials.gov NCT03659877, retrospectively registered on September 6th 2018.

## Background

Maintenance of adequate physical activity (PA) is a key lifestyle recommendation for many chronic disease populations, including heart failure (HF) patients, with benefits including improvements in exercise capacity, health-related quality-of-life and reduced all-cause and HF-specific mortality and hospital admissions [1–4]. Progressively, clinical trials are relying on accelerometers to objectively measure levels of PA and inactivity, to investigate the relationship between PA, inactivity and HF disease progression [5], or to evaluate the effect of a PA or exercise programme in primary or secondary prevention in HF [6]. However, there is yet to be consensus on the most appropriate way to convert raw acceleration data into behaviourally relevant metrics, particularly in chronic disease populations.

International PA recommendations for public health and cardiac patients are based on time spent in moderate-to-vigorous PA (MVPA) [7, 8]. In order to derive information on the amount of time spent in different PA intensities from accelerometers, cut-points or intensity thresholds derived from calibration studies are applied to the raw data. Previous studies measuring PA and inactivity patterns in HF patients have used intensity thresholds based on calibration studies involving young, healthy individuals rather than HF-specific populations [9–12]. Applying these thresholds to HF patients assumes the energy cost for a given activity is the same for everyone, which may lead researchers to misclassify PA levels of people with HF due to the lack of consideration for an individual’s exercise tolerance [13, 14]. Prince et al. [15] have shown that application of multiple published thresholds resulted in widely varying interpretations of PA levels in patients with coronary artery disease, emphasising the need for caution when deciding which thresholds to use in clinical populations. Furthermore, some commercially available PA monitors use privately owned, proprietary algorithms to transform the raw data into units of activity. This can complicate interpretation of results both in clinical and non-clinical populations where these devices have not been validated for use in research, but also across studies using different brands of accelerometer or PA monitor. Improving the way in which PA is measured in HF patients will allow for better monitoring, classification and treatment allocation of HF patients. Therefore recent publications have called for population specific calibration studies [13, 14].

We conducted a laboratory-based calibration study aiming to estimate the acceleration values for hip- and wrist-worn accelerometers which correspond to both inactivity and MVPA in patients with HF via calibration with oxygen consumption (VO_2_). A secondary aim was to compare the derived thresholds to current generic intensity thresholds. We hypothesized that the derived accelerometer thresholds would be lower than the generic thresholds based on calibration studies of healthy adults, due to the reduced exercise capacity and breathlessness, dominant symptoms of HF.

## Methods

### Study design

This was a single centre, observational study of a cohort of HF patients. The design was based on previous calibration studies [11, 12, 16].

### Participants

A sample of 22 adults with HF were recruited from the Royal Devon and Exeter NHS Foundation Trust HF clinic between March 2018 and October 2018. Inclusion criteria were adult (≥18 years) outpatients with a diagnosis of HF confirmed by a hospital specialist, New York Heart Association (NYHA) class I to III symptoms, who were able to give informed consent. The exclusion criteria were: acute decompensated HF, contraindication to exercise testing or PA, resident in a long term care facility, unwilling or unable to travel to the research site, patients unable to understand the study information, and judged unable to participate for any other reason.

The study protocol conforms to the 1975 Declaration of Helsinki, ethical approval was granted by Cambridge South Research Ethics Committee (18/EE/0019), and the trial registration ID is: NCT03659877. Participants gave informed consent prior to data being collected.

### Activities

Participants attended the sports science laboratory at University of Exeter St Luke’s campus in the UK. They were asked to take their medication as normal, and to not eat or drink caffeinated or calorie containing foods or drinks prior to the visit which was scheduled in the morning. This fasting period was required to avoid error in resting metabolic rate (RMR) measurement associated with increased metabolic rate with digestion, absorption and metabolism of dietary nutrients. Breakfast was provided after RMR measurement, prior to any physical activities being performed. The laboratory protocol consisted of a series of activities (listed in order of completion, Table 1), chosen based on previous calibration studies [11, 12, 16] and selected with the help of a local HF patient and public involvement group to be representative of daily living activities for HF patients. The duration of each activity was chosen to optimise the likelihood of steady state metabolism being achieved.

**Table 1:** List of activities performed in order, their duration, and associated notes

Activity	Duration	Notes
Laying down on a bed	30 min	Patients laid down in low- or semi-Fowler’s position (as per patient preference) [17]. RMR was directly measured during minutes 10–20.
Sitting on the bed	5 min	
ISWT	Performed until stopping criteria met	
Sitting watching TV	5 min	
Standing washing and drying dishes	5 min	
Sitting quietly	5 min	
Walking at a pace perceived to be light	3–5 min*	Pace derived from ISWT (RPE 11)
Walking at a pace perceived to be moderate	3–5 min*	Pace derived from ISWT (RPE 13)
Light pace walk carrying 2 × 1.5 kg shopping bags	3–5 min*	Pace derived from ISWT (RPE 11)

* Patients unable to complete 5 min walking did a minimum of 3 min to optimise the likelihood of steady state metabolism being achieved. *RMR* resting metabolic rate, *ISWT* incremental shuttle walk test, *RPE* rating of perceived exertion

ISWT was performed to measure exercise capacity and gauge rating of perceived exertion (RPE) over the completed stages, which informed the light (RPE 11) and moderate (RPE 13) walking paces. Standardised instructions were given prior to the test, and no encouragement given throughout [18, 19]. Stopping criteria were when the participant was too breathless to continue, unable to maintain the required speed, or they decided to stop. At the end of the ISWT and each walking activity, participants were asked to sit and rest quietly until they felt ready to complete the next task. Other activities had a 1 min transition period. Participants that used walking aids in their daily life, were allowed to do so throughout the activities as required. The patient visit lasted approximately 3 h in total, most of which was administrative or resting time, with up to 45 min of physical tasks.

### Measures

#### Anthropometric measures

Prior to the activities, weight was measured, to the nearest 0.1 kg using an electronic scale (Seca, Hamburg, Germany), height was measured to the nearest 0.1 cm using a stadiometer (Seca, Hamburg, Germany), both without shoes. Blood pressure was assessed using a manual sphygmomanometer (Accoson, England). Body mass index was also calculated.

#### Oxygen consumption (VO_2_)

VO_2_ was measured throughout each activity with a portable Oxycon mobile breath-by-breath ergospirometry system, (VIASYS Healthcare GmbH, Hoechburg, Germany). This system has been shown to be a reliable and valid method of measuring energy expenditure [20]. Standardised gas and volume calibration was performed within 1 h before each participant visit according to manufacturer’s specifications [21]. The flow meter was calibrated automatically. VO_2_ was expressed in millilitres per kilogram per minute (ml/kg/min).

#### Accelerometry

Throughout each activity, participants wore 3 GENEActiv accelerometers (Activinsights, Kimbolton, UK); one on each wrist, secured using a watch strap; and on the waist, secured using an elasticated waist band over the left iliac crest. Acceleration was measured between -8 *g* and 8 *g*, and raw triaxial acceleration recorded at 100 Hz. The GENEActiv accelerometer has been validated for both hip- and wrist-worn measurement of PA, and to distinguish between inactivity, light PA, and MVPA in healthy adults [22].

#### Rating of perceived exertion

Before starting any activities, participants were instructed on how to use the Borg 15 point RPE scale [23], which was reported during the last 30 s of each ISWT level and during the last minute of all other activities.

### Data reduction

Immediately after testing was completed, the accelerometers and ergorespirometry system were removed and data were downloaded to a personal computer.

#### Oxygen consumption data

VO_2_ was averaged over minutes 10–20 of lying down, and over the last minute of all other activities. VO_2_ data for each individual for each activity was converted into metabolic equivalents (METs) in two different ways; standard METs calculated using the standard formula (VO_2_/3.5 ml/kg/min), and measured METs using each individual participants measured RMR (VO_2_/RMR). For the purpose of comparison to the general population, predicted METs for each activity were taken from the compendium of physical activities [24]. Activities with METs ≥3.0 were considered MVPA, and < 1.5 METs as inactivity. Sedentary time is defined as a combination of sitting or reclining and low energy expenditure during waking hours [25]. The time spent below 1.5 METs measured by wrist worn accelerometry can only measure inactivity, and not the specific posture required to be defined as sedentary time, therefore we use the term inactivity.

#### Accelerometer data

GENEActiv data were downloaded using GENEActiv PC software (version 3.2; Activinsights, Kimbolton, Cambridge, UK) and averaged over 5 s epochs, which is considered adequate for reporting different activities [26].

Each axis (x, y, z) of the raw tri-axial data was multiplied by 1000 to transform the signals from *g* to milligravity units (m*g*), to ensure the subsequent accelerometer thresholds would be comparable to prior literature. Raw tri-axial data were then summarized into a single vector magnitude using three common approaches:

(1) gravity-subtracted sum of vector magnitudes (SVM) (eq. 1), where the vector magnitude is calculated in each epoch and 1000 mg is subtracted, when the accelerometer is static and the earth’s gravitation pull is the only acceleration, the result is 0 [22].

1 $$ SVM=\frac{1}{n}\times \sum \mid \sqrt{\Big({x}^2+{y}^2+{z}^2}\Big)-1000\mathrm{m}g\mid $$

(2) mean amplitude deviation (MAD) (eq. 2), which describes the typical distance of data points around the mean [27].

2 $$ MAD=\frac{1}{n}\times \sum \mid {r}_i-\overline{r\mid } $$

Where;

$$ {r}_i=\sqrt{x_i^2+{y}_i^2+{z}_i^2} $$

$$ \overline{r}= mean\ vector\ magnitude\ within\ the\ time\ period\ of\ interest $$

(3) Euclidean Norm Minus One (ENMO) (eq. 3), where vector magnitude is calculated in each epoch, 1000 mg is subtracted, and negative ENMO values rounded to 0 [28].

3 $$ ENMO=\sqrt{\left({x}^2+{y}^2+{z}^2\right)}-1000\mathrm{m}g $$

During our data processing it was observed that at low magnitude of acceleration, ENMO returned a high frequency of 0’s, making it severely limited in classifying inactivity and light PA. This observation has also been identified in another study [29]. Therefore ENMO was excluded from further analysis.

In line with the VO_2_ data, MAD and SVM values were averaged over minutes 10–20 of lying down, and the last minute of all other activities.

### Data analysis

Conservatively assuming a ROC AUC of 0.85 (based on lowest AUC previously reported [11, 12, 16], and assumed null AUC of 0.5 (no association) at 90% power and 5% alpha, a minimum sample size of 18 patients was required.

#### Initial data checks

Repeated measures correlations (r_RM_) were calculated to establish the presence and strength of the within-participant association between both accelerometer measures (SVM and MAD) and the measured METs as a necessary precursor to applying ROC curve analysis and prediction models to establish accelerometer cut-points for 1.5 and 3.0 METs. This was achieved by utilising the sum of squares (SS) values from an ‘analysis of covariance’ model with METs as the outcome variable, participant number as the independent factor variable, and the accelerometer measure as the continuous covariate: r_RM_ = SQRT [covariate_SS_ / (covariate_SS_ + residual_SS_)] [30]. The r_RM_ values were interpreted according to Cohen’s effect size i.e. weak, r = 0.1 to 0.29; moderate r = 0.3 to 0.49; strong r ≥ 0.5.

#### Intensity threshold derivation

Based on methods used in previous calibration studies [11, 12, 16] we used a combination of receiver-operator characteristic (ROC) analysis and mixed effects regression model analysis methods to establish accelerometer thresholds for inactivity (< 1.5 METs) and MVPA (≥3 METs). A different threshold was produced for each combination of ‘data analysis method’ by ‘body location’ by ‘data reduction method’ separately. The robustness of each data analysis and data reduction technique were taken into account in order to decide which accelerometer thresholds should be recommended for future use.

ROC analysis was performed using the ‘roctab’ and ‘roccomp’ STATA commands. The continuous measured MET values were coded into the following intensity categories: inactivity (< 1.5 METs: yes/no), MVPA (≥3.0 METs: yes/no) to create binary indicators. The mg values that maximised the combination of sensitivity and specificity were selected as the threshold values. AUC values for each ROC curve calculated were defined as excellent (≥0.90), good (0.80–0.89), fair (0.70–0.79), poor (0.60–0.69) or failure (< 0.60).

Multilevel mixed effects linear regression modelled the accelerometer-derived mg values across the range of measured METs achieved during the different activities. This analysis was performed using the ‘xtmixed’ STATA command where METs was entered into the model as both a fixed and random effect. This allowed the ‘mg against METS’ slopes and intercepts to vary between individuals. The resulting regression equation was used to calculate intensity thresholds for inactivity (< 1.5 METs) and MVPA (≥3.0 METs).

#### Sensitivity analysis

Sensitivity analyses were undertaken by repeating the ROC analysis and multilevel mixed effects linear regression excluding participants that used a walking aid, as this may have affected accelerometer readings, and excluding washing up as an activity, as this involves high levels of wrist movement, but little waist movement to explore the impact on the resulting accelerometer thresholds and model fits.

#### Validation analysis

In order to validate the derived intensity thresholds (via multilevel mixed effects linear regression) a leave-one-out cross validation method was used. One observation was left out and used as the test dataset, and a multilevel mixed effects linear regression model was fitted and used to predict the left out observation, this was then repeated sequentially for all possible observations. A median split of the actual acceleration values and the predicted values were cross-tabulated to obtain a ‘percentage of correct predictions’.

Statistical analyses were performed using Stata (V.15.0; StataCorp, College Station, Texas, USA). Leave-one-out cross validation analysis was performed using the R programming language and environment (V3.6.1). All data are expressed as mean values and standard deviations unless otherwise stated. The level of significance was set at *p* < 0.05.

## Results

Table 2 details the characteristics of the study participants.

**Table 2:** Patient characteristics

Characteristic	*N* = 22 patients Mean ± SD unless otherwise stated
Male (n, %)	17 (77)
Age (years)	70.7 ± 14.1
Body Mass Index (kg/m^2^)	28.1 ± 4.4
LVEF (%)	34.5 ± 14.0
Reduced LVEF < 40% (*n*, %)	14 (64)
Mid-range LVEF 40–49% (*n*, %)	4 (18)
Preserved EF ≥50% (*n*, %)	4 (18)
NYHA class (*n*, %)	
I	1 (4)
II	18 (82)
III	3 (14)
IV	0
Dilated cardiomyopathy (*n*, %)	14 (64)
Ischaemic heart disease (*n*, %)	8 (36)
ICD/CRT/Pacemaker (*n*, %)	13 (59)
ACE-I/ARB/ARNI (*n*, %)	21 (95)
Beta-blocker (*n*, %)	22 (100)
MRA (*n*, %)	14 (64)
Loop diuretic (*n*, %)	17 (77)
Hypertension (*n*, %)	11 (50)
Diabetes (*n*, %)	6 (27)
COPD (*n*, %)	4 (18)
Arthritis (*n*, %)	2 (9)
AF (*n*, %)	11 (50)
Stroke (*n*, %)	5 (23)
Comorbidities (hypertension, diabetes, COPD, arthritis, AF, stroke) (*n*, %)	
0 comorbidity	6 (27)
1 comorbidity	4 (18)
2 comorbidities	5 (23)
3 comorbidities	4 (18)
4+ comorbidities	3 (14)
ISWT distance (m)	286.4 ± 190.6
RMR (mL O_2_·kg^−1^·min^−1^)	2.67 ± 0.66

Data presented as mean ± standard deviation or as number (percentage). *LVEF* left ventricular ejection fraction, *NYHA* New York Heart Association, *ICD* implantable cardioverter defibrillator, *CRT* cardiac resynchronisation therapy, *ACE-I* angiotensin-converting enzyme inhibitor, *ARB* angiotensin receptor blocker, *ARNI* angiotensin II receptor blocker neprilysin inhibitor, *MRA* mineralocorticoid receptor antagonist, *COPD* chronic obstructive pulmonary disease, *AF* atrial fibrillation, *ISWT* incremental shuttle walk test

### Accelerometry and METS

All participants completed all activities within the study protocol. Data from the ISWT were not included in the threshold generation analysis due to the small numbers of participants that reached the latter stages. All three accelerometers failed to record for one participant so they were omitted from further analysis. For a second participant, the left wrist accelerometer failed to record, so only their right wrist and waist data was included.

Accelerometer outputs (SVM and MAD), METs (standard, measured and predicted), and RPE scores for each of the activities are reported in Table 3. For all activities, measured METs ranged 33–35% higher than standard METs. Similarly, measured METs ranged 7–105% higher than the compendium predicted METs [24].

**Table 3:** Mean (SD) accelerometer values, METS, and RPE for each activity

Physical activity	Accelerometer values: SVM			Accelerometer values: MAD			METS (measured RMR)^*^ (*N* = 21)	METS (standard RMR)^†^ (*N* = 21)	Predicted METS^‡^ (*N* = 21)	RPE score (*N* = 21)
	Right wrist (m*g*) (*N* = 21)	Left wrist (m*g*) (*N* = 20)	Waist (m*g*) (*N* = 21)	Right wrist (m*g*) (*N* = 21)	Left wrist (m*g*) (*N* = 20)	Waist (m*g*) (*N* = 21)				
Laying down	4.7 (1.9)	4.8 (2.3)	3.7 (0.8)	3.3 (4.7)	2.1 (4.2)	0.4 (0.3)	1.0 (0.0)	0.8 (0.2)	1.0	6.5 (1.2)
Sitting (fasted)	7.3 (2.7)	8.7 (4.8)	4.3 (0.9)	13.0 (19.9)	13.8 (13.6)	0.6 (0.5)	1.2 (0.2)	0.9 (0.2)	1.3	6.5 (1.1)
Sitting watching TV	8.4 (3.2)	6.6 (2.8)	4.1 (0.9)	14.3 (14.1)	10.4 (12.2)	0.9 (1.5)	1.3 (0.2)	1.0 (0.2)	1.3	6.4 (0.8)
Standing washing & drying dishes	74.4 (22.4)	55.1 (18.9)	8.7 (2.5)	54.3 (24.7)	45.4 (16.7)	2.0 (2.7)	2.6 (0.5)	1.9 (0.4)	1.8	8.9 (2.3)
Sitting quietly	9.8 (4.8)	14.4 (14.1)	4.3 (1.0)	18.5 (24.0)	34.8 (35.0)	0.8 (0.7)	1.4 (0.2)	1.1 (0.3)	1.3	7.1 (1.7)
Light pace walk (average pace 1.6 mph)	57.8 (17.3)	62.6 (25.1)	62.9 (28.8)	27.9 (23.3)	24.4 (23.3)	3.2 (2.5)	4.1 (1.0)	3.0 (0.6)	2.0	10.6 (2.2)
Moderate pace walk (average pace 2.2 mph)	79.2 (27.3)	76.9 (31.1)	86.0 (40.2)	34.1 (23.9)	26.7 (17.0)	4.3 (2.5)	4.7 (1.1)	3.5 (0.8)	2.8	12.9 (1.5)
Light pace walk carrying shopping bags (2 × 1.5 kg) (average pace 1.6 mph)	55.5 (20.7)	56.9 (20.9)	66.5 (29.8)	24.7 (33.2)	16.4 (27.3)	3.3 (1.4)	4.4 (0.9)	3.2 (0.6)	2.5	13 (2.5)

*SVM* sum of vector magnitude, *MAD* mean amplitude deviation, *METS* metabolic equivalents, *RPE* rating of perceived exertion, *ISWT* incremental shuttle walk test

*Measured METS = VO2 (ml/kg/min) measured during each activity / VO2 (ml/kg/min) measured at rest (resting metabolic rate).

†Standard METS = VO2 (ml/kg/min) measured during each activity / 3.5 (ml/kg/min).

‡Predicted METS taken from most similar activity in the compendium of physical activity

**Fig. 1:**
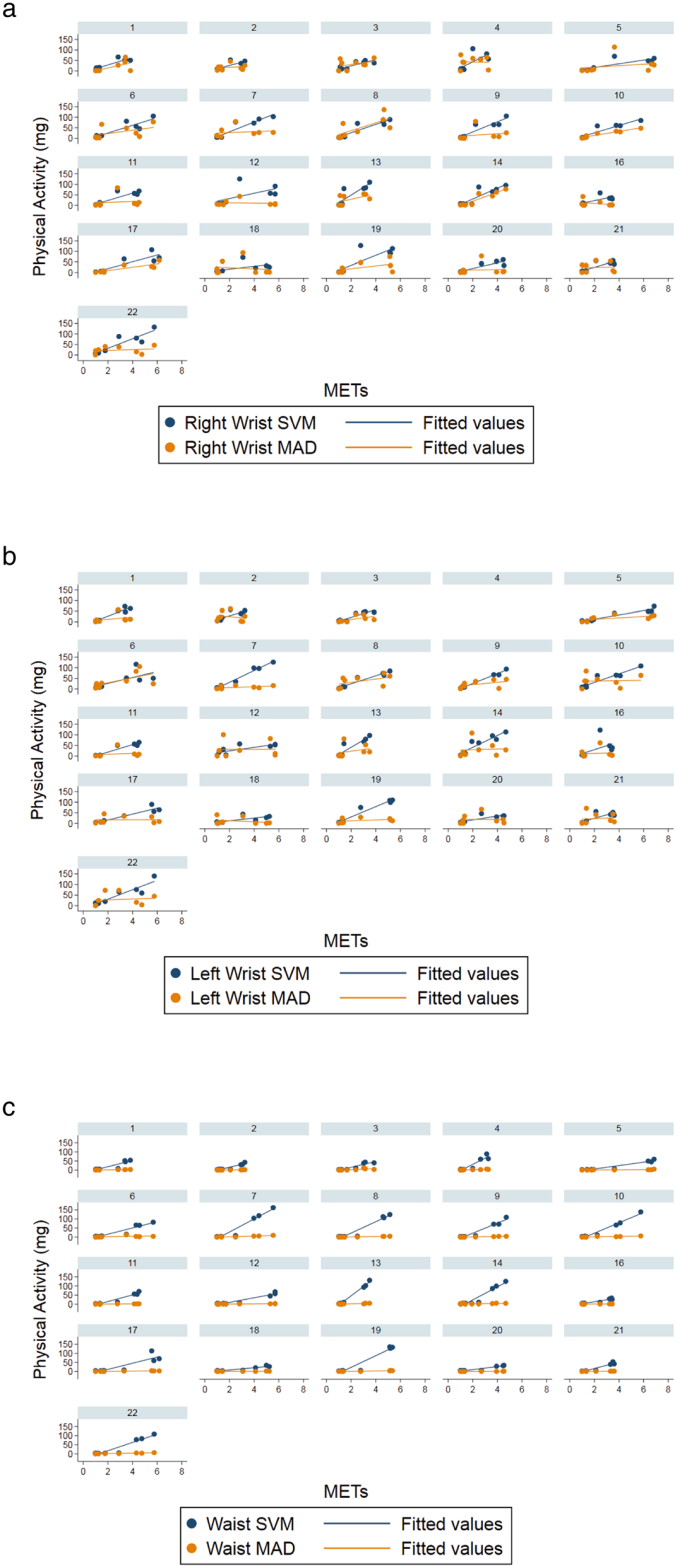
Trellis plot showing acceleration values in mg vs intensity in METs for each activity and fitted regression lines, for SVM (blue) and MAD (orange), for **a** right wrist, **b** left wrist, **c** waist worn accelerometers

Figure 1 shows the relationships between SVM and measured METs and MAD and measured METs for each participant at each accelerometer wear location. Accelerometer values increased in line with the increase in METs. There was a strong correlation between SVM and METs (left wrist r_RM_ = 0.84, right wrist r_RM_ = 0.80, waist r_RM_ = 0.86, all *p* < 0.001). The correlation was weak between left wrist MAD and METs (r_RM_ = 0.19, *p* = 0.026), moderate between right wrist MAD and METs (r_RM_ = 0.33, p < 0.001), and strong between waist MAD and METs (r_RM_ = 0.67, p < 0.001).

### ROC curve analysis

ROC analysis results are presented in Additional file 1. GENEActiv accelerometers at all locations were able to discriminate between inactivity, and MVPA. SVM gave more precise discrimination across all three accelerometer wear locations, and both inactivity and MVPA (AUC = 0.93 to 0.99) compared to MAD (AUC = 0.61 to 0.97). All derived inactivity and MVPA thresholds were lower than the commonly used thresholds previously published at all wear locations [11, 12].

### Multilevel mixed effects regression analysis

Table 4 shows the multilevel mixed effects regression model coefficients and constants, and the derived inactivity and MVPA intensity thresholds calculated by inputting 1.5 METs and 3.0 METs respectively. All derived thresholds for inactivity were much lower than the threshold for inactivity of 45.8 mg commonly applied to all populations [12]. Right wrist: SVM = 13.3–18.6 mg, MAD = 14.2–18.4 mg. Left wrist: SVM = 14.4–16.9 mg, MAD = 15.4–18.8 mg. Waist: SVM = 7.6–11.1 mg, MAD = 1.0 mg.

**Table 4:** Mixed effect regression models and resultant inactivity and MVPA intensity thresholds for SVM and MAD

	Coefficient (95% CI)	Constant (95%CI)	Inactivity Threshold (< 1.5 METs) (m***g***) (95% CI)†	MVPA Threshold (≥3.0 METs) (m***g***) (95% CI)†
*SVM*				
*Right wrist*				
All patients (*n* = 21, obs = 168)	17.9 (15.4 to 20.5)***	−8.3 (−14.2 to −2.3)**	18.6 (8.8 to 28.4)	45.5 (31.9 to 59.1)
Excluded aided walking activity data‡ (*n* = 21, obs = 159)	20.2 (17.6 to 22.9)***	−11.7 (− 17.6 to −5.9)***	18.6 (8.7 to 28.5)	49.0 (35.1 to 62.9)
Excluded aided walking activity data and washing up activity data §(*n* = 21, obs = 138)	19.8 (17.7 to 22.0)***	−16.4 (− 19.9 to − 13.0)***	13.3 (6.7 to 19.9)	43.1 (33.3 to 52.9)
*Left wrist*				
All patients (*n* = 20, obs = 160)	18.0 (15.5 to 20.5)***	−10.3 (− 15.4 to −5.2)***	16.7 (7.8 to 25.6)	43.6 (38.5 to 56.3)
Excluded aided walking activity data‡ (*n* = 20, obs = 151)	20.1 (17.7 to 22.5)***	−13.2 (− 18.2 to −8.2)***	16.9 (8.3 to 25.5)	47.0 (34.8 to 59.2)
Excluded aided walking activity data and washing up activity data § (*n* = 20, obs = 131)	19.9 (17.6 to 22.2)***	−15.5 (− 19.6 to − 11.5)***	14.4 (6.8 to 21.85)	44.3 (33.2to 55.2)
*Waist*				
All patients (*n* = 21, obs = 168)	22.0 (18.3 to 25.7)***	−25.4 (−30.5 to − 20.2)***	7.6 (−3.1 to 18.4)	40.6 (24.3 to 57.0)
Excluded aided walking activity data‡ (*n* = 20, obs = 159)	22.9 (19.6 to 26.2)***	−26.6 (−31.7 to − 21.5)***	7.7 (− 2.3 to 17.8)	42.0 (27.1 to 57.0)
Excluded aided walking activity data and washing up activity data § (*n* = 21, obs = 138)	24.0 (20.2 to 27.9)***	−24.9 (− 29.5 to − 20.4)***	11.1 (0.78 to 21.49)	47.2 (31.0 to 63.32)
*MAD*				
*Right wrist*				
All patients (*n* = 21, obs = 168)	5.3 (2.5 to 8.0)***	10.4 (3.1 to 17.8)**	18.3 (6.9 to 29.7)	26.2 (10.7 to 41.7)
Excluded aided walking activity data‡ (*n* = 21, obs = 159)	7.4 (4.5 to 10.3)***	7.3 (−0.1 to 14.6)	18.4 (6.6 to 30.1)	29.5 (13.4 to 45.6)
Excluded aided walking activity data and washing up activity data § (*n* = 21, obs = 138)	7.0 (4.2 to 9.8)***	3.7 (−2.6 to 10.0)	14.2 (3.6 to 24.8)	24.7 (9.9 to 39.5)
*Left wrist*				
All patients (*n* = 20, obs = 160)	2.8 (0.3 to 5.2)*	14.6 (7.5 to 21.7)***	18.7 (7.9 to 29.5)	22.8 (8.3 to 37.3)
Excluded aided walking activity data‡ (*n* = 20, obs = 151)	3.7 (1.0 to 6.3)**	13.3 (6.1 to 20.5)***	18.8 (7.6 to 29.9)	24.2 (9.1 to 39.4)
Excluded aided walking activity data and washing up activity data § (*n* = 20, obs = 131)	3.5 (0.8 to 6.2)*	10.1 (3.2 to 17.0)**	15.4 (4.4 to 26.3)	20.7 (5.7 to 35.6)
*Waist*				
All patients (*n* = 21, obs = 168)	0.9 (0.7 to 1.2)***	−0.5 (−0.9 to 0.0)	1.0 (0.2 to 1.7)	2.4 (1.3 to 3.5)
Excluded aided walking activity data‡ (*n* = 21, obs = 159)	1.0 (0.8 to 1.2)***	−0.6 (−1.1 to − 0.1)*	1.0 (0.2 to 1.8)	2.5 (1.4 to 3.6)
Excluded aided walking activity data and washing up activity data § (*n* = 21, obs = 138)	1.1 (0.8 to 1.3)***	−0.6 (−1.0 to − 0.2)**	1.0 (0.3 to 1.7)	2.6 (1.5 to 3.6)

*MVPA* moderate-to-vigorous physical activity, *SVM* sum of vector magnitudes, *MAD* mean amplitude deviation, *METS* metabolic equivalents

**†**95% CI calculated using upper and lower bounds of coefficient and constant in formula.

* *p* < 0.05, ** *p* < 0.01, *** *p* < 0.001

‡ Excluded walking activity data for *n* = 3 patients using walking aids.

§ Excluded walking activity data for *n* = 3 patients using walking aids and all washing up activity data

Crucially, even the highest HF-derived MVPA threshold (49 mg) is much lower than the MVPA threshold of 93.2 mg or 100 mg that are commonly applied to all populations [11]. Right wrist: SVM = 43.1-49 mg, MAD = 24.7–29.5 mg. Left wrist: SVM = 43.6–47.0 mg, MAD = 20.7–24.2 mg. Waist: SVM = 40.6–47.2 mg, MAD = 2.4–2.6 mg. MVPA thresholds did not differ by location (wrist or waist) for SVM, but were much lower at the waist compared to the wrist for MAD.

### Sensitivity analysis

Excluding aided walking activity data during the ROC analysis made slight differences to the AUC (differences ranging from − 0.01 to 0.04%), and made small differences to the derived thresholds (differences ranging from − 5.7 to 5.3 mg) across all accelerometer wear locations and data reduction methods (additional file 1). Excluding walking data of patients who used walking aids, plus all washing and drying dishes data during the ROC analysis made little difference to the AUC (differences ranging from − 0.2 to 0.11%), and threshold differences ranged from − 5.7 to 20.4 mg (additional file 1).

In the multilevel mixed effects regression analysis, excluding aided walking activity data made minimal difference to the inactivity thresholds (differences ranging from 0 to 0.2 mg), and MVPA thresholds (differences ranging from 0.1 to 3.5 mg) across all accelerometer wear locations and data reduction methods (Table 4). Similarly, when excluding walking data of patients who used walking aids, plus all washing and drying dishes data during the multilevel mixed effects regression analysis were minimal for inactivity (differences ranging from − 5.3 to 3.5 mg) and MVPA (differences ranging − 2.4 to 6.6 mg) across all accelerometer wear locations and data reduction methods (Table 4).

### Validation analysis

Leave-one-out cross validation (Additional file 2) of the multilevel models showed that the model fit for SVM at each wear location was acceptable but appeared to under predict at high PA and MET levels. Proportion of correct predictions were high (right wrist: 96%; left wrist: 99%, waist: 95%). Models using MAD performed less well with lower proportions of correct predictions (right wrist: 69%; left wrist: 64%; waist: 87%).

Comparing the robustness and goodness of fit across all the data reduction methods, data analysis methods and the resulting models, the best fit for the data appeared to be when using SVM data reduction and multilevel mixed effects regression analysis with all data.

## Discussion

The aim of this study was to estimate the hip- and wrist-worn accelerometer values which correspond to inactivity and MVPA in HF patients. This is the first study to derive HF specific accelerometer intensity thresholds for time spent inactive and in MVPA. Intensity thresholds corresponding to inactivity were much lower than those previously published based on young healthy adults [12]. Although less investigated than MVPA, inactivity thresholds of < 50 mg or < 40 mg have been previously proposed for GENEActiv accelerometers [12, 31, 32]. This suggests the possibility that researchers using generic intensity thresholds are concluding that HF patients are more inactive when they may actually be engaging in light intensity activities.

Similarly, accelerometer thresholds corresponding to MVPA were much lower than those derived from other calibration studies both in healthy adults and older adults [11, 14]. Applying intensity thresholds developed in younger, healthier populations to HF patients assumes the energy cost for a given activity is the same for everyone, with no consideration for an individual’s exercise capacity [13]. In line with previous studies, we showed HF patients require greater energy expenditure to complete walking and self-paced daily living activities, where METs calculated using measured RMR were higher than METs calculated using the standard RMR estimate of 3.5 ml/kg/min [33–36]. Additionally, measured METs were higher than the predicted METs from the compendium of physical activities [17]. Often, self-reported PA measures use the compendium to inform activity estimates and it is also used to prescribe PA [37, 38]. Our study clearly highlights the limitations of using standard RMR values, and existing MET tables to estimate the time HF patients spend in MVPA.

The average measured RMR for this sample of HF patients was 2.67 ml/kg/min, 24% less than the standard 3.5 ml/kg/min, consistent with RMRs reported previously in older adults [33], and HF patients [35]. The application of standard RMR for MET calculations is common, including in previous calibration studies [11, 12], however several studies have shown the inaccuracy of using estimated RMR in elderly and clinical populations including HF patients [33–35]. Although the mechanism for decreased RMR in HF patients compared to healthy individuals is currently unknown, decline with increasing NYHA class has been shown, and may be influenced by changes in skeletal muscle physiology associated with a reduced cardiac output in HF [35]. It may be argued that our lower RMR is due, to some extent, to being measured whilst supine, rather than sitting, however we measured supine RMR in line with current best practices [17].

### Data reduction and analysis techniques

We explored 3 data reduction approaches for generating a single value of acceleration from the x, y, z axes. We found that ENMO returned a high frequency of 0’s across all activities, which has also been observed by others [29], and therefore excluded it from further analysis. SVM had stronger correlations with METS, produced higher AUC values in the ROC analysis, and returned better model fit predictions in the leave-one-out cross validation analysis compared to MAD.

ROC analysis was less robust than multilevel mixed effect regression analysis when using MAD, with poor-fair AUC for wrist accelerometers. This may be due to the dichotomisation of MET data in the ROC analysis, which leads to a loss of statistical power, whereas the absolute MET values are used in the multilevel mixed effect regression analysis. Furthermore, the multilevel mixed effect regression correctly accounts for the clustering of measures within individuals which ROC analysis does not.

Therefore we recommend studies measuring PA levels in HF patients with accelerometers use the thresholds derived using SVM and multilevel mixed effect regression for all patients, i.e. inactivity (right wrist: 18.6 mg, left wrist: 16.7 mg, waist: 7.6 mg) and MVPA (right wrist: 45.5 mg, left wrist: 43.6, waist: 40.6 mg).

### Strengths and limitations

Strengths of this study include the use of both wrist- and waist-worn accelerometers with known reliability and validity, comparison of multiple data reduction algorithms, and comprehensive data analysis of raw acceleration data captured at a high sampling frequency. We were thus able to generate HF specific intensity thresholds, enabling more accurate differentiation between inactivity, and MVPA behaviours of HF patients. In contrast to previous calibration studies we have individually measured RMR, and used this to more accurately measure METs for each activity for each individual [11, 12]. We selected representative HF patients from a hospital clinic, who were heterogeneous in exercise capacity and age, factors known to affect PA measurement [13, 14, 32], and determined activities with the assistance of a HF Patient and Public Involvement group to represent typical daily living, with the majority of PA and exercise from walking and household activities.

We recognise that this study has some limitations. It was based on small, single-centre sample of HF patients, therefore we are unable to determine how the thresholds may vary between NYHA classes or sex, for example. In addition, it is difficult to determine whether HF medications taken by the patients (100% patients taking β-blockers) influenced VO_2_ or heart rate. The PAs were undertaken in laboratory conditions rather than free-living which also limits the generalisability of the result. Attempting to apply a single threshold to all within a population may not be possible since individual capacities vary [39]. Employing a threshold or cut-point technique to derive PA metrics from accelerometers may not be as accurate as newer techniques such as machine learning that are being explored in public health studies [40, 41]. However, whilst PA recommendations are based on classes of PA intensity rather than specific behaviours, these techniques are still pragmatic to use until consensus is reached.

### Implications and future research

We have developed a new approach that better captures PA in HF patients using accelerometry. Our results suggest that application of previously published intensity thresholds based on calibration studies of adults without chronic disease potentially risks underestimation and misclassification of PA in HF patients. Larger studies, using our approach are now required to clarify PA levels in the various severity levels of HF, taking account of comorbidity. We suggest power calculations should take into account the small numbers of patients that reach the latter stages of the ISWT to ensure spread of patients fitness levels represented.

This study also has important implications for PA and exercise prescription. It is vital both the patient and the clinician are aware of the PAs that will count as MVPA and benefit the patient, as prescribing activities that are too intense may lead to decreased motivation and adherence to PA guidelines or cardiac rehabilitation [42]. Our results show that any walking activity, including at a slow speed, would be sufficient for HF patients to accumulate minutes of MVPA. Researchers should avoid applying accelerometer thresholds, estimated MET values from look-up tables or standard RMR values, which are based on healthy populations to patients with HF, and refer to studies such as ours where the specific clinical population has been studied.

## Conclusions

HF specific accelerometer intensity thresholds for both inactivity and MVPA were substantially lower (< 50%) than previously published and commonly used intensity thresholds. Using cut-points or intensity thresholds based on calibration studies of younger, healthy adults assumes energy expenditure is the same for everyone, regardless of an individual’s exercise capacity. We demonstrated that HF patients had measured RMR values which were 24% lower than the standardised value of 3.5 ml/kg/min, and require more energy to perform typical daily living activities, including walking and household activities, with higher measured MET values compared to METs calculated using assumed RMR, or METs predicted from the compendium of physical activities. We thereby demonstrate that the application of generic PA thresholds may result in a misclassification and underestimation of the true amount of MVPA undertaken by HF patients.

## Supplementary information

**Additional file 1.**

**Additional file 2.**

## Data Availability

The datasets used and/or analysed during the current study are available from the corresponding author on reasonable request.

## Abbreviations

ENMO: Euclidean norm minus one
HF: Heart failure
ISWT: Incremental shuttle walk test
MAD: Mean amplitude deviation
METs: Metabolic equivalents
MVPA: Moderate-to-vigorous physical activity
NYHA: New York Heart Association
PA: Physical activity
RMR: Resting metabolic rate
ROC: Receiver-operator characteristic
RPE: Rating of perceived exertion
SVM: Sum of vector magnitude
VO_2_: Oxygen consumption
