# Supplementary material for: Physical activity assessment by accelerometry in people with heart failure
Source: BMC Sports Sci Med Rehabil. 2020 Aug 12;12:47. doi: 10.1186/s13102-020-00196-7 (PMC7425563; doi:10.1186/s13102-020-00196-7)
Supplement: Supplementary file 1 — Additional file 1. [file 13102_2020_196_MOESM1_ESM.pdf]

Additional file 1

Table A: Sensitivity, specificity, area under the curve and resultant cut points for each accelerometer, and each data reduction method

	**Inactivity (<1.5 METs)**				**MVPA (>3.0 METs)**			
	**Sensitivity (%)**	**Specificity (%)**	**AUC (95% CI)**	**Threshold (mg)**	**Sensitivity (%)**	**Specificity (%)**	**AUC (95% CI)**	**Threshold (mg)**
SVM Right Wrist								
All patients (n=21, obs =168)	94.5	96.1	0.96 (0.93-0.99)	15.2	89.2	85.5	0.90 (0.85-0.95)	35.7
Excluded aided walking activity data* (n=21, obs=159)	91.5	98.7	0.96 (0.92 to 0.99)	20.5	100.0	81.6	0.91 (0.85 to 0.95)	30.0
Excluded aided walking and washing up activity data† data (n=21, obs=138)	91.9	97.4	0.96 (0.92-1.00)	15.2	100.0	97.7	1.00 (0.98-1.00)	30.0
SVM Left wrist								

All patients (n=20, obs =160)	92.0	91.8	0.96 (0.92-0.99)	16.9	84.1	85.6	0.91 (0.87-0.96)	39.5
Excluded aided walking activity data* (n=21, obs=151)	92.3	94.5	0.96 (0.92-0.99)	19.8	96.3	83.5	0.93 (0.89-0.97)	36.4
Excluded aided walking and washing up activity data† (n=21, obs=131)	88.1	98.6	0.95 (0.91-1.00)	29.9	98.0	97.5	0.99 (0.98-1.00)	36.4
SVM Waist								
All patients (n=21, obs =168)	90.1	90.9	0.95 (0.92-0.98)	5.6	95.4	98.1	0.99 (0.98-1.00)	16.2
Excluded aided walking activity data* (n=21, obs=159)	86.6	97.4	0.94 (0.91-0.98)	6.1	94.6	98.1	0.99 (0.98-1.00)	16.2

Excluded aided walking and washing up activity data† (n=21, obs=138)	87.1	100.0	0.93 (0.89-0.98)	26.0	100.0	97.7	1.00 (0.99-1.00)	26.0
MAD Right wrist								
All patients (n=21, obs =168)	75.8	63.6	0.76 (0.69-0.84)	6.7	58.5	68.9	0.67 (0.59-0.75)	22.4
Excluded aided walking activity data* (n=21, obs=159)	82.9	63.6	0.79 (0.72-0.86)	6.7	67.9	68.9	0.71 (0.63-0.79)	22.4
Excluded aided walking and washing up activity data† (n=21, obs=138)	77.4	64.5	0.76 (0.68-0.84)	6.7	65.4	81.4	0.78 (0.70-0.85)	22.4
MAD Left wrist								
All patients (n=21, obs =168)	77.0	63.0	0.73 (0.65-0.81)	7.7	74.6	51.6	0.61 (0.52-0.69)	7.7

Excluded aided walking activity data* (n=21, obs=159)	80.8	63.0	0.75 (0.67-0.83)	7.7	79.6	51.6	0.63 (0.54-0.72)	7.7
Excluded aided walking and washing up activity data† (n=21, obs=138)	74.6	63.9	0.72 (0.63-0.80)	7.7	78.0	61.7	0.71 (0.62-0.80)	7.7
MAD Waist								
All patients (n=21, obs =168)	83.5	87.0	0.90 (0.85-0.95)	1.0	90.8	86.4	0.94 (0.90-0.97)	1.5
Excluded aided walking activity data* (n=21, obs=159)	84.2	87.0	0.90 (0.85-0.95)	1.0	92.9	86.4	0.95 (0.92-0.98)	1.6
Excluded aided walking and washing up activity data† (n=21, obs=138)	87.1	90.8	0.91 (0.85-0.97)	1.2	94.2	91.9	0.97 (0.94-1.00)	1.6

METS, metabolic equivalents; MVPA, moderate-vigorous physical activity; AUC, area under the curve; SVM, sum of vector magnitude; MAD, mean amplitude deviation.* Excluded walking activity data for n=3 patients using walking aids. † Excluded walking activity data for n=3 patients using walking aids and all washing up activity data.
