# Supplementary material for: Physical activity assessment by accelerometry in people with heart failure
Source: BMC Sports Sci Med Rehabil. 2020 Aug 12;12:47. doi: 10.1186/s13102-020-00196-7 (PMC7425563; doi:10.1186/s13102-020-00196-7)
Supplement: Supplementary file 2 — Additional file 2. [file 13102_2020_196_MOESM2_ESM.pdf]

Additional file 2 – Leave-one-out cross validation

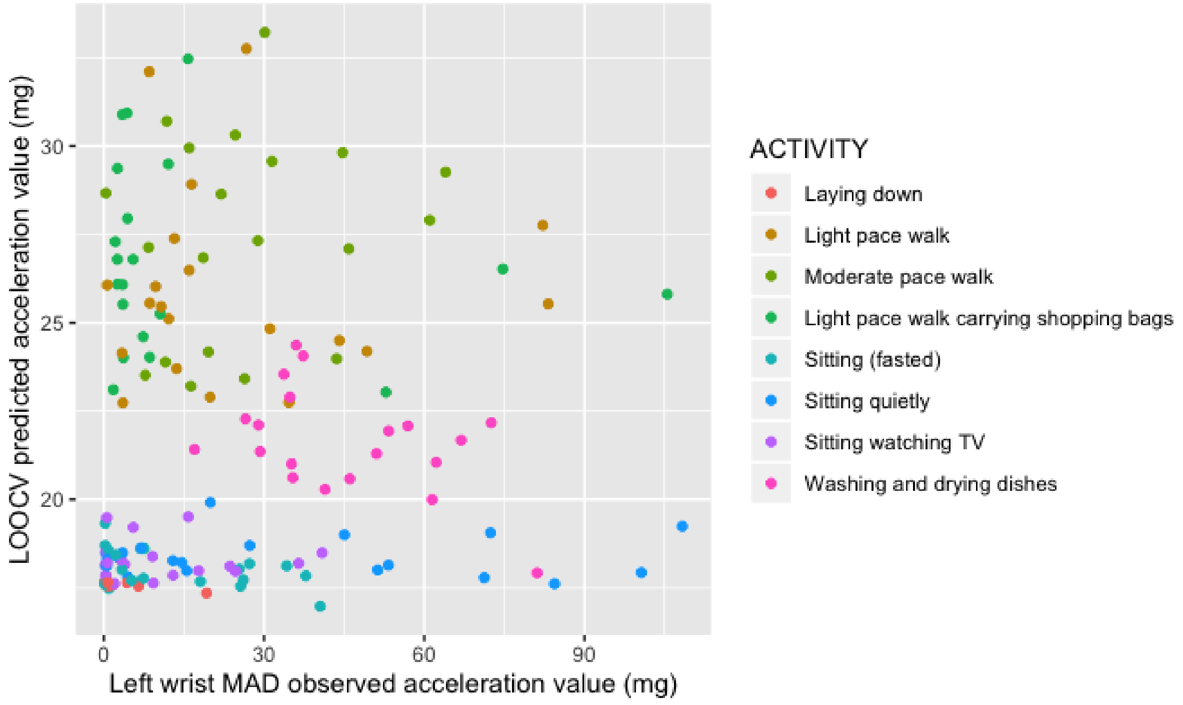

Figure B1 – Scatter plot where each point represents the prediction from a multilevel mixed effects regression linear regression model leaving out one observation against the observed left wrist MAD acceleration value, shaded by activity.

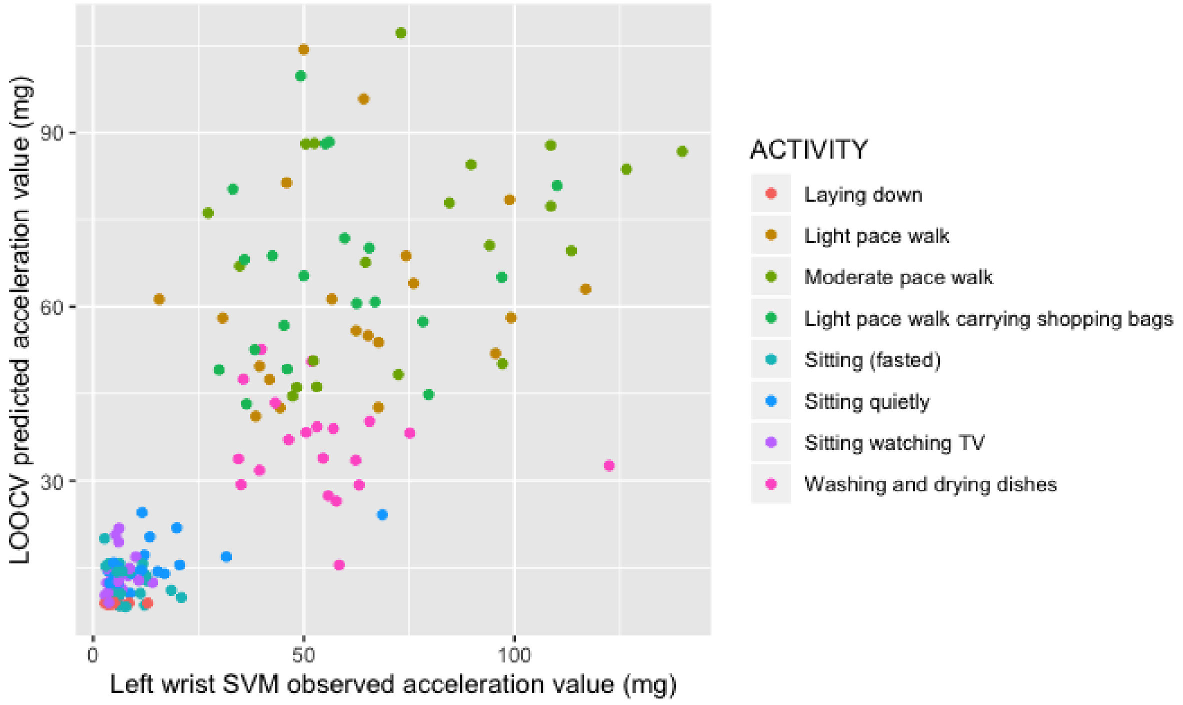

Figure B2 - Scatter plot where each point represents the prediction from a multilevel mixed effects regression linear regression model leaving out one observation against the observed left wrist SVM acceleration value, shaded by activity.

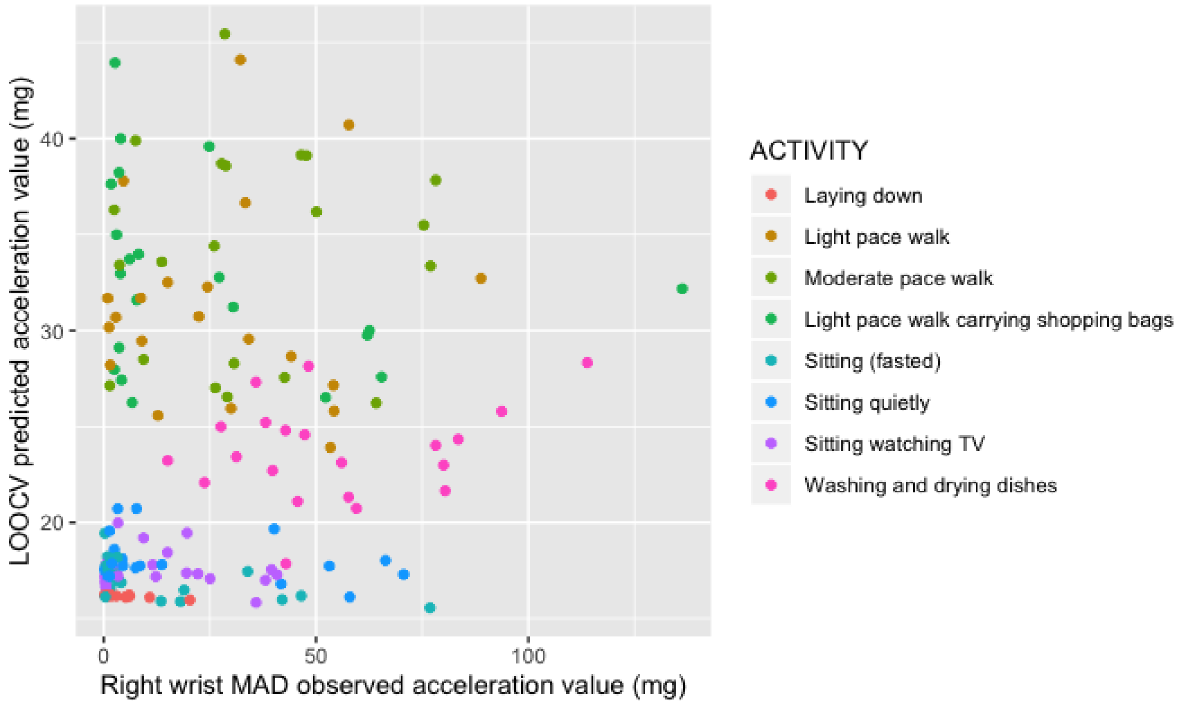

Figure B3 - Scatter plot where each point represents the prediction from a multilevel mixed effects regression linear regression model leaving out one observation against the observed right wrist MAD acceleration value, shaded by activity.

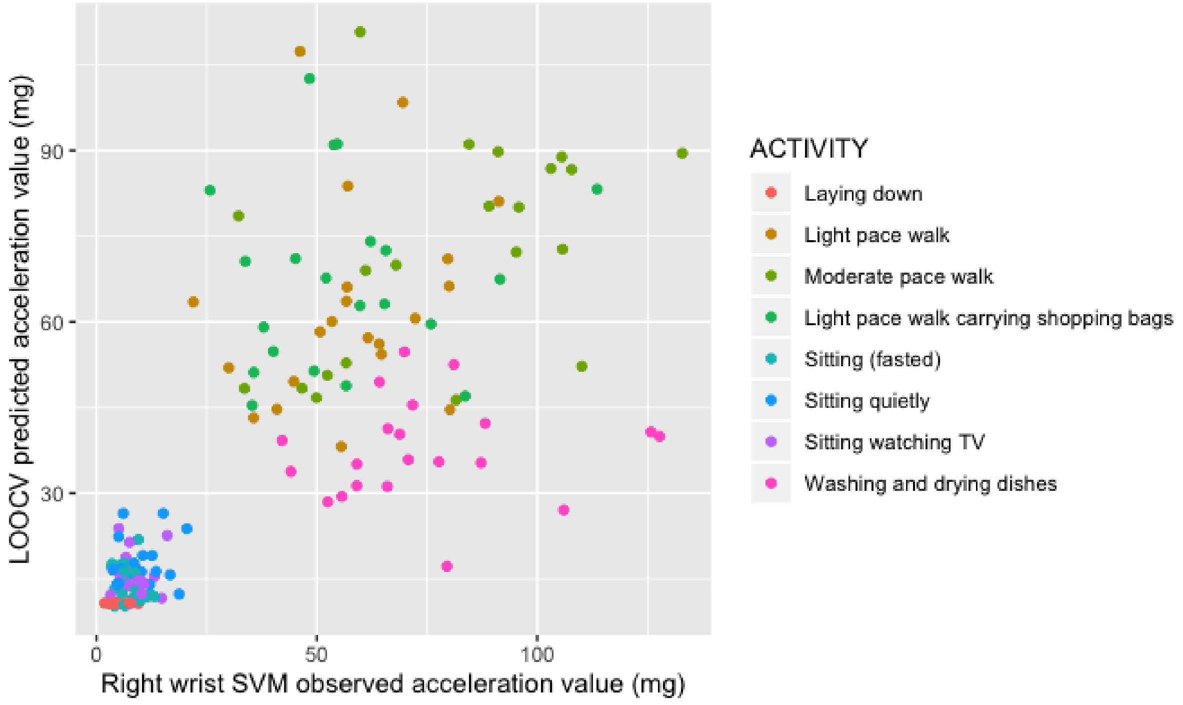

Figure B4 - Scatter plot where each point represents the prediction from a multilevel mixed effects regression linear regression model leaving out one observation against the observed right wrist SVM acceleration value, shaded by activity.

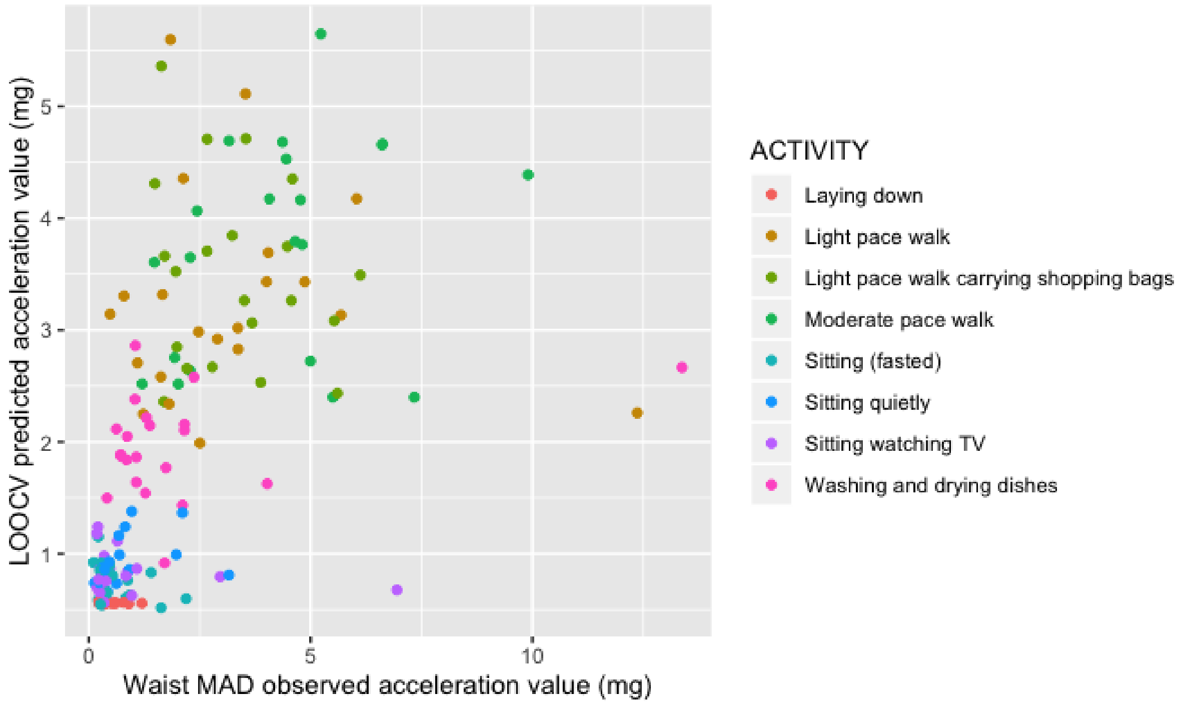

Figure B5 - Scatter plot where each point represents the prediction from a multilevel mixed effects regression linear regression model leaving out one observation against the observed waist MAD acceleration value, shaded by activity.

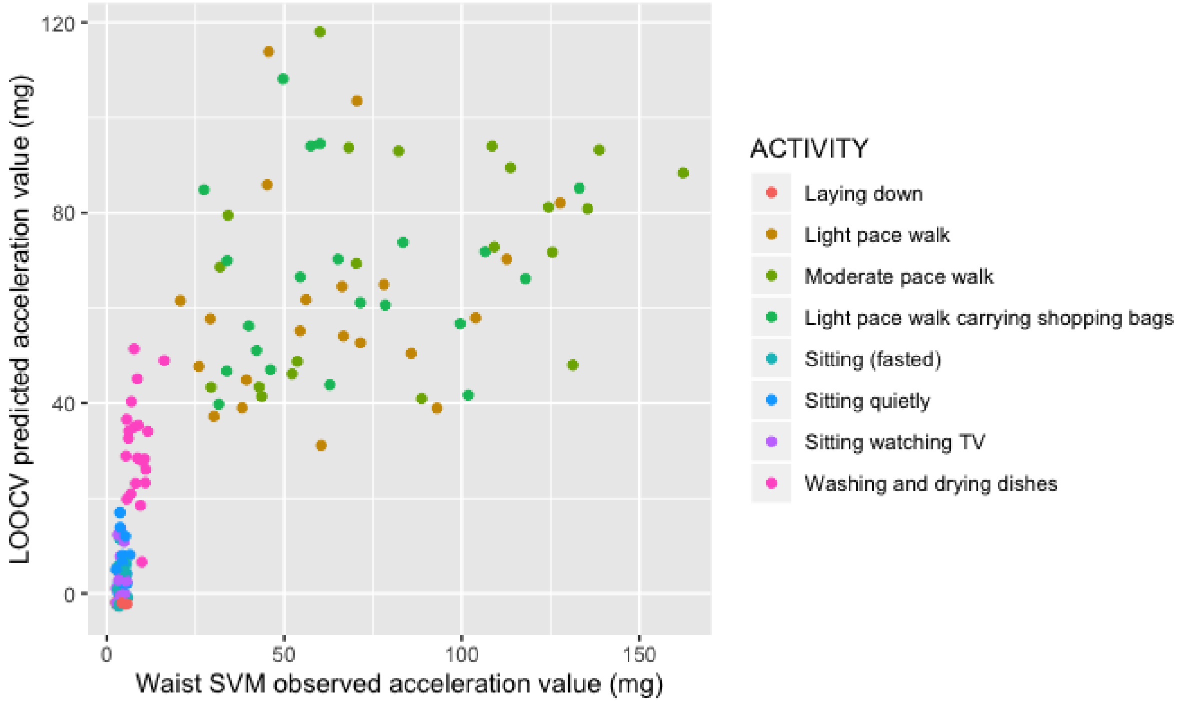

Figure B6 - Scatter plot where each point represents the prediction from a multilevel mixed effects regression linear regression model leaving out one observation against the observed waist SVM acceleration value, shaded by activity.
